# Upregulation of nuclear protein Hemgn by transcriptional repressor Gfi1 through repressing PU.1 contributes to the anti-apoptotic activity of Gfi1

**DOI:** 10.1016/j.jbc.2024.107860

**Published:** 2024-10-05

**Authors:** Binod G C, Laney Jia Hoyt, Sinisa Dovat, Fan Dong

**Affiliations:** 1 Department of Biological Sciences, University of Toledo, Toledo, Ohio, USA; 2 Department of Pediatrics, Pennsylvania State University College of Medicine, Hershey, Pennsylvania, USA

**Keywords:** apoptosis, cell death, histone demethylase, transcription, transcription repressor, Gfi1, PU.1, Ikaros, Hemgn

## Abstract

Gfi1 is a transcriptional repressor that plays a critical role in hematopoiesis. The repressive activity of Gfi1 is mediated mainly by its SNAG domain that interacts with and thereby recruits the histone demethylase LSD1 to its target genes. An important function of Gfi1 is to protect hematopoietic cells against stress-induced apoptosis, which has been attributed to its participation in the posttranscriptional modifications of p53 protein, leading to suppression of p53 activity. In this study, we show that Gfi1 upregulated the expression of Hemgn, a nuclear protein, through a 16-bp promoter region spanning from +47 to +63 bp relative to the transcription start site (TSS), which was dependent on its interaction with LSD1. We further demonstrate that Gfi1, Ikaros, and PU.1 are bound to this 16-bp region. However, while Ikaros activated *Hemgn* and collaborated with Gfi1 to augment Hemgn expression, it was not required for Gfi1-mediated Hemgn upregulation. In contrast, PU.1 repressed *Hemgn* and inhibited Hemgn upregulation by Gfi1. Notably, PU.1 knockdown and deficiency, while augmenting Hemgn expression, abolished Hemgn upregulation by Gfi1. *PU.1* (*Spi-1*) is repressed by Gfi1. We show here that *PU.1* repression by Gfi1 preceded and correlated well with Hemgn upregulation. Thus, our data strongly suggest that Gfi1 upregulates Hemgn by repressing *PU.1*. In addition, we demonstrate that Hemgn upregulation contributed to the anti-apoptotic activity of Gfi1 in a p53-independent manner.

Growth factor independence 1 (Gfi1) is a zinc-finger transcriptional repressor critically involved in hematopoiesis (1). Gfi1 is required for maintaining the quiescence and self-renewal capacity of hematopoietic stem cells (HSCs) and controls the development of HSCs into lymphoid and myeloid cells (2, 3, 4). Gfi1 deficiency significantly reduces the population of common lymphoid progenitors (CLP) and impairs T- and B-cell differentiation (5, 6). In the myeloid lineage, Gfi1 promotes neutrophil development at the expense of monocyte formation. Targeted deletion of *Gfi1* in mice blocks neutrophil development leading to severe neutropenia accompanied by an expansion of atypical monocytes (7, 8, 9). As a transcriptional repressor, Gfi1 has been shown to repress genes that are key regulators of hematopoiesis, primarily by recruiting the histone demethylase LSD1 to the promoters of its target genes through its SNAG domain (10, 11).

Gfi1 has an important role in protecting hematopoietic cells against apoptosis. Overexpression of Gfi1 in T and B cells has been shown to abolish G1 cell cycle arrest and apoptosis induced by growth factor withdrawal (12, 13, 14). Targeted deletion of Gfi1 increases apoptosis in T-cell precursors and peripheral mature T-cells (15, 16). HSCs, T lymphoid, and myeloid precursors from *Gfi1*^*−/−*^ mice are hypersensitive to stress-induced apoptosis (4). Gfi1 exerts its anti-apoptosis activity in part *via* its involvement in post-translational modifications of the C-terminal domain of the p53 protein, which inhibits p53 activity (17). We and others have shown that Gfi1 inhibited apoptosis through p53-independnt mechanisms (18, 19). Indeed, it has been shown recently that Gfi1 may inhibit apoptosis through modulation of sphingolipid metabolism by repressing *SGPP1* in multiple myeloma (MM) cells and by regulating PRMT1-dependent methylation of proteins involved in DNA repair such as MRE11 and 53BP1, which is necessary for their function (20, 21).

Hemgn, also known as embryonic development-associated gene (EDAG) in humans, is a nuclear protein that is primarily expressed in HSCs and early progenitor cells (22, 23). HSCs from *Hemgn*^*−/−*^ mice are functionally impaired, displaying defective engraftment activity in competitive repopulation assay that is associated with increased apoptosis and enhanced IFN-γ response (24). Conversely, Hemgn overexpression in human and mouse hematopoietic progenitor cells enhances their proliferation, survival, and self-renewal, which promotes their expansion in mice (25, 26, 27, 28, 29). When overexpressed, Hemgn also favors myelopoiesis over lymphopoiesis in mice (30). *Hemgn* is transcriptionally activated by HOXB4 and GATA1, and its expression is induced in response to DNA damage (24, 31, 32, 33). Significantly, overexpression of Hemgn has been observed in acute myeloid leukemia (AML) and acute lymphoblastic leukemia (ALL) and may confer resistance to chemotherapy (26, 27, 28, 29).

In this study, we show that Gfi1 upregulated Hemgn expression through the 16-bp region (+47/+63 bp relative to TSS) in the *Hemgn* promoter, which was dependent on its interaction with LSD1. We further demonstrate that Gfi1, Ikaros, and PU.1 are bound to this 16-bp region. Notably, Ikaros activated *Hemgn*, but was not required for Hemgn upregulation by Gfi1. In contrast, PU.1 repressed *Hemgn*, and its knockdown or deficiency abolished Hemgn upregulation. Gfi1 has been shown to repress *PU.1* (34). We show that *PU.1* repression by Gfi1 preceded and correlated well with Hemgn upregulation. Together, these data strongly suggest that Gfi1 upregulates Hemgn by repressing *PU.1*. In addition, we demonstrate that Hemgn upregulation contributed to the anti-apoptotic activity of Gfi1 in a p53-independent manner.

## Results

### Gfi1 upregulates Hemgn expression

**Figure 1:**
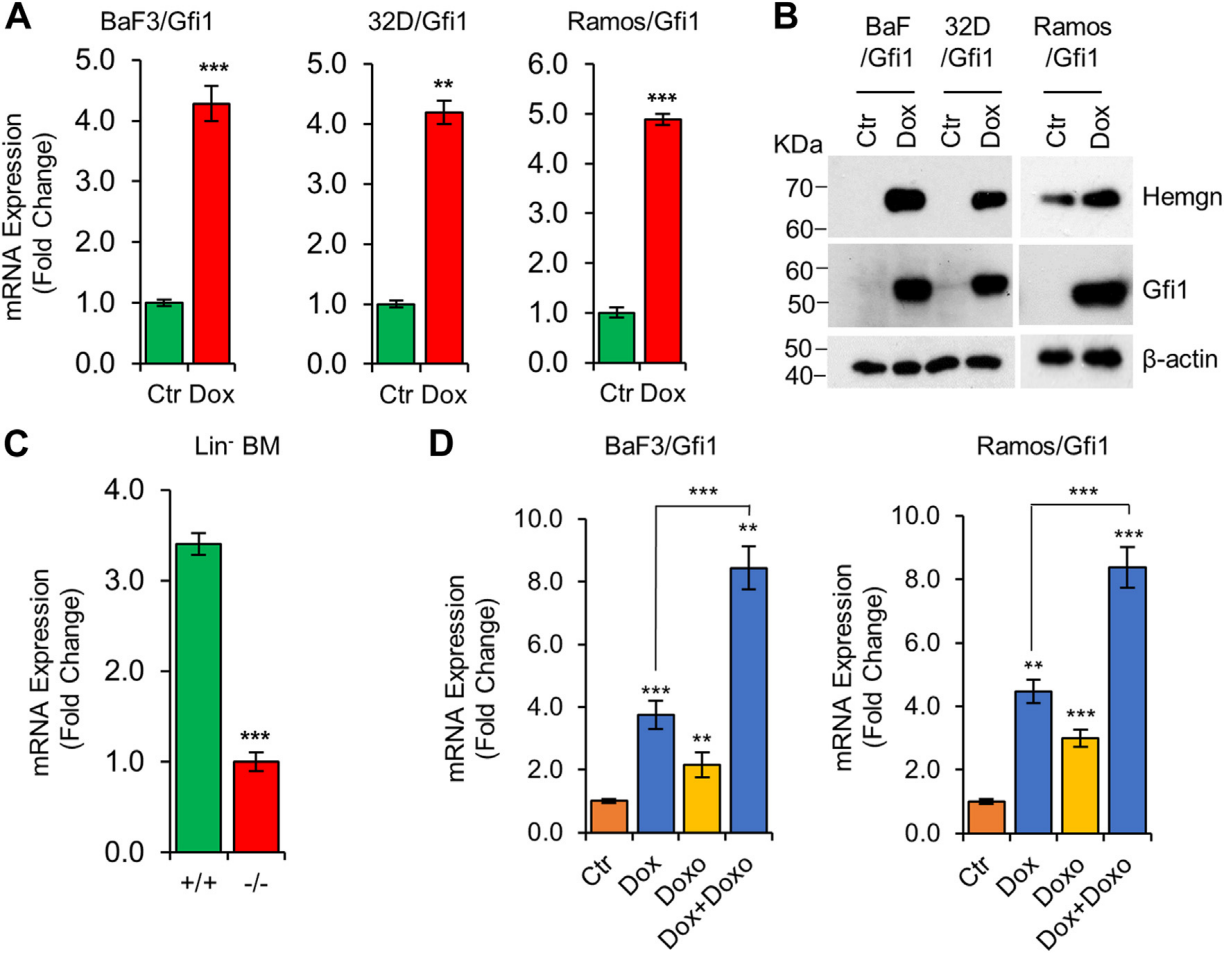
**Gfi1 upregulates Hemgn expression.***A* and *B*, cells as indicated were left untreated (Ctr) or treated with Dox (1 μg/ml) for 24 h, followed by evaluation of Hemgn mRNA and protein expression by qRT-PCR (*A*) and Western blot analysis (*B*). *C*, Hemgn mRNA levels were analyzed in Lin^-^ BM cells from *Gfi1*^*+/+*^ and *Gfi1*^*−/−*^ mice. *D*, BaF/Gfi1 (*left panel*) and Ramos/Gfi1 (*right panel*) cells were left untreated (Ctr) or treated with Dox for 6 h and then incubated with or without Doxo for 16 h prior to evaluation of Hemgn mRNA levels. Data are presented as mean ± SD (N = 3). Statistically significant differences in Hemgn expression levels: ∗∗*p* < 0.01; ∗∗∗*p* < 0.001.

Although Gfi1 is known as a transcriptional repressor, we were interested to know whether Gfi1 may also activate the expression of certain genes. Gene chip microarray was employed to assess the gene expression profile in the murine pro-B BaF3 cells transduced with a doxycycline (Dox)-inducible lentiviral expression construct encoding Gfi1 (BaF/Gfi1) (18). We observed the upregulation of several genes, notably including Hemgn, in BaF/Gfi1 cells in which Gfi1 expression was induced by the addition of Dox. Upregulation of Hemgn upon Dox induction was confirmed subsequently in BaF/Gfi1 cells by quantitative real-time RT-PCR (qRT-PCR) and Western blot analysis (Fig.1, *A* and *B*). We next examined whether Gfi1 upregulated Hemgn in murine myeloid 32D and human Burkitt's lymphoma Ramos cells transduced with the Dox-inducible Gfi1-expressing lentiviral construct (18, 35). As shown in Fig. 1, *A* and *B*, Hemgn mRNA and protein levels were significantly increased in Dox-treated 32D/Gfi1 and Ramos/Gfi1 cells. In contrast, Hemgn mRNA expression was significantly reduced in the Lin^-^ bone marrow (BM) cells from *Gfi1*^*−/−*^ mice as compared to *Gfi1*^*+/+*^ BM cells (Fig. 1*C*). Hemgn expression has been shown to be induced by DNA damage (24, 36). We further examined whether Gfi1 influenced Hemgn expression in response to DNA damage. BaF/Gfi1 and Ramos/Gfi1 cells were first treated with Dox to induce Gfi1 expression, followed by treatment with doxorubicin (Doxo) to induce DNA damage. As shown in Fig. 1*D*, Hemgn expression was augmented in response to Doxo treatment in both cell lines and treatment with a combination of Dox and Doxo led to further increases in Hemgn expression. Collectively, these results established that Gfi1 upregulates Hemgn expression in mouse and human hematopoietic cells. As Ramos cells lack functional p53, the results obtained in Ramos cells also indicated that Hemgn upregulation by Gfi1 and DNA damage was independent of p53.

### LSD1 interaction is required for Gfi1-mediated Hemgn upregulation

**Figure 2:**
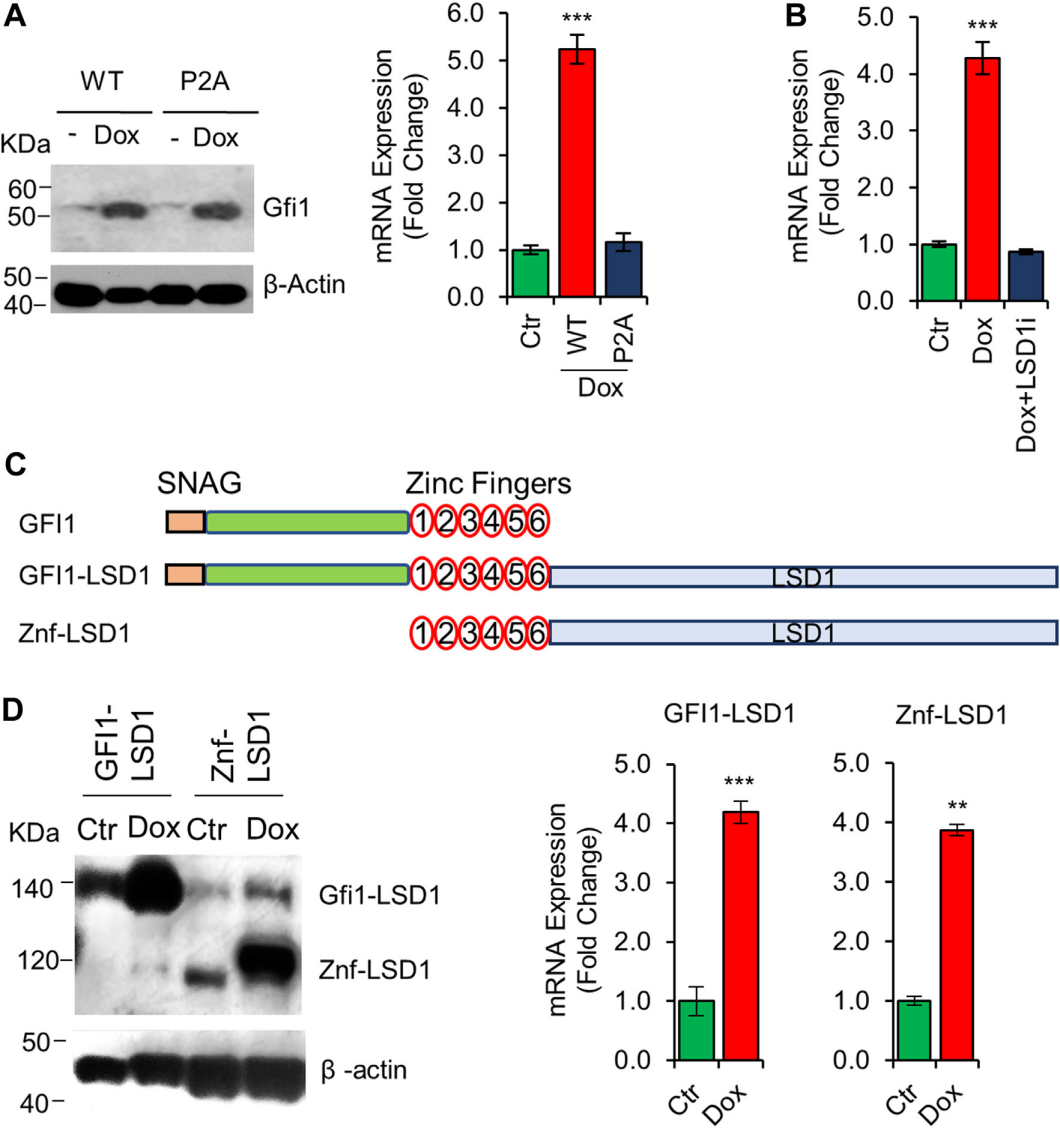
**LSD1 is required for Gfi1-mediated Hemgn upregulation.***A*, BaF/Gfi1 and BaF/Gfi1P2A cells were *left* untreated or treated with Dox (1 μg/ml) for 24 h and examined for expression of Gfi1 proteins (*left panel*) and Hemgn mRNA (*right panel*). *B*, BaF/Gfi1 cells were treated with Dox overnight and then with LSD1i (1 μM) for 24 h prior to evaluation of Hemgn mRNA levels. *C*, schematic diagrams of Gfi1-LSD1 and Znf-LSD1 fusion proteins. *D*, BaF3 cells expressing the Dox-inducible fusion proteins as indicated were *left* untreated or treated with Dox for 24 h and then examined for expression of the fusion proteins using the anti-Flag antibody (*left panel*) and Hemgn mRNA (*right panel*). Data are shown as mean ± SD (n = 3). Statistical significances: ∗∗*p* < 0.01; ∗∗∗*p* < 0.001.

It has been shown that the SNAG domain of Gfi1 is required for Gfi1-mediated transcriptional repression by interacting with LSD1. A P2A point mutation in the SNAG domain impedes Gfi1 interaction with LSD1, rendering it inactive as a transcriptional repressor (1, 37). We examined the impact of the P2A mutation on Gfi1-mediated Hemgn upregulation in BaF3 cells transduced with the Dox-inducible Gfi1 P2A mutant (Fig. 2*A*). Gfi1-mediated Hemgn upregulation was abolished by the P2A mutation, suggesting that Hemgn upregulation by Gfi1 relied on its capability to recruit LSD1 *via* the SNAG domain. We further investigated the effect of an LSD1 inhibitor GSK2879552 (LSD1i), which inhibits LSD1 histone demethylase activity, on Gfi1-mediated Hemgn upregulation. As shown in Fig. 2*B*, LSD1i treatment effectively suppressed Hemgn upregulation by Gfi1 in BaF/Gfi1 cells.

We sought to examine if LSD1 interaction alone is sufficient for Gfi1-mediated Hemgn upregulation. To address this, BaF3 cells were transduced with the Dox-inducible lentiviral constructs encoding two different fusion proteins consisting of LSD1 fused to either the full-length Gfi1 (Gfi1-LSD1) or only the 6 C-terminal zinc finger domains that are required for DNA binding (ZNF-LSD1) (Fig. 2*C*). The expression of the fusion proteins upon Dox induction was confirmed by Western blotting analysis (Fig. 2*D*). Notably, Gfi1-LSD1 and ZNF-LSD1 were equally capable of upregulating Hemgn expression upon induction of their expression with Dox. Together, these results indicated that LSD1 interaction is essential and sufficient for Gfi1-mediated upregulation of Hemgn.

### A 16-bp sequence of *Hemgn* spanning from +47 to +63 bp is required for Gfi1-mediated upregulation

**Figure 3:**
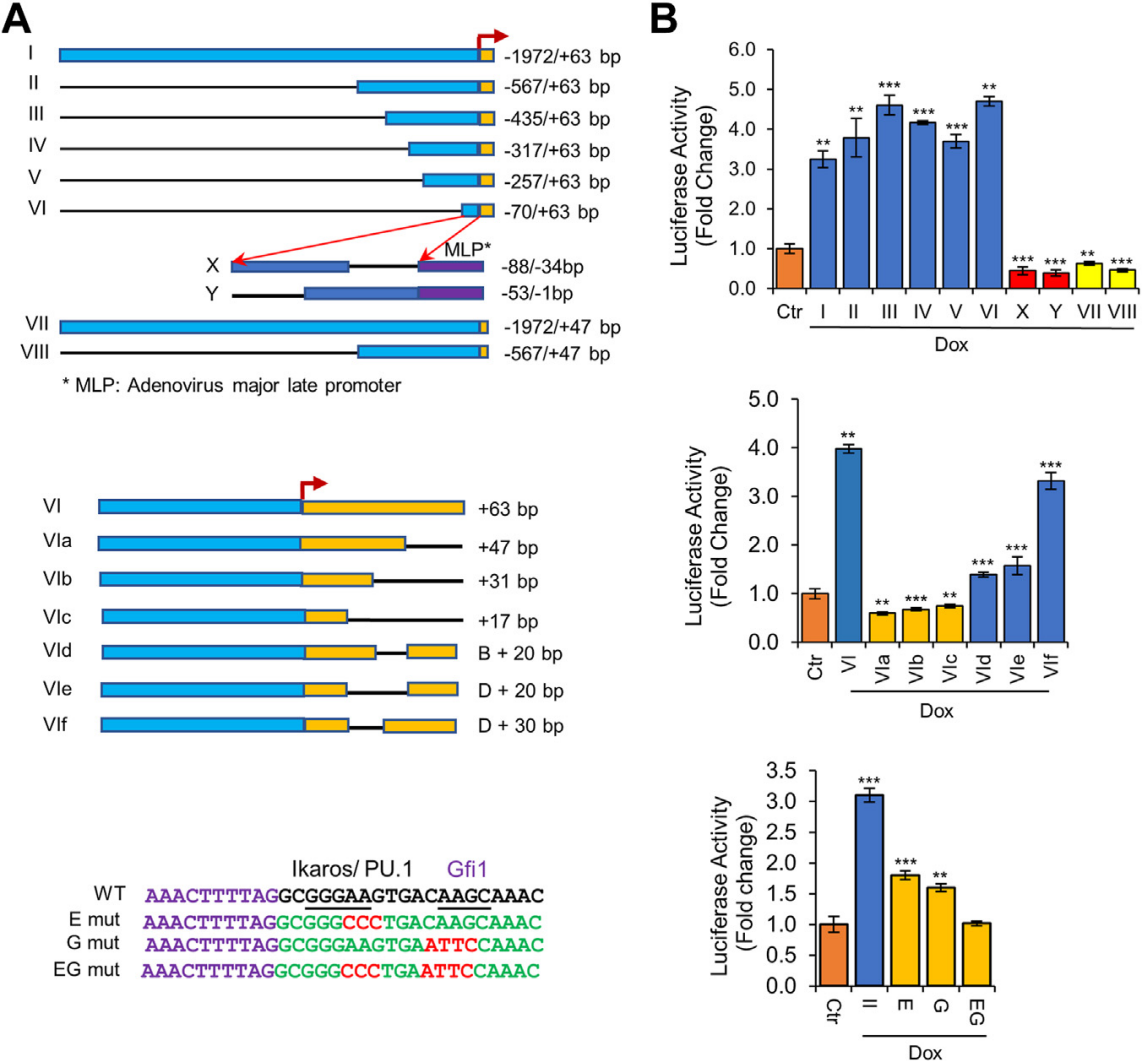
**The 16-bp region spanning from +47 to +63 bp of *Hemgn* is essential for Gfi1-mediated Hemgn upregulation.***A*, schematic diagrams of the various mouse *Hemgn* promoter fragments (*upper panel*) and the different deletion mutants derived from fragment VI (*middle panel*), and the nucleotide sequence of *Hemgn* promoter spanning from +33 to +63 bp and the mutations introduced in this region of fragment II. *B*, the reporter constructs containing the different promoter fragments were transfected into BaF/Gfi1 cells, followed by treatment with Dox (1 μg/ml) for 24 h. Promoter activation, shown as fold changes as compared to untreated cells, was determined. Data are shown as mean ± SD (n = 3). ∗∗*p* < 0.01; ∗∗∗*p* < 0.001.

We next investigated whether Gfi1 activated the *Hemgn* promoter. Consistent with upregulation of Hemgn mRNA expression, Gfi1 activated the *Hemgn* promoter fragment spanning from −1972 to +63 bp in luciferase reporter assay (fragment I; Fig. 3*A*). Progressive truncation of the *Hemgn* promoter fragment from the 5′ end revealed that a fragment spanning from −70 to +63 bp (fragment VI) was sufficient for its activation by Gfi1 (Fig. 3*B*). However, Gfi1 failed to activate the *Hemgn* promoter fragments spanning from −88 to −34 bp and −51 to −1 bp that were placed upstream of the adenovirus minimal promoter MLP (major late promoter), suggesting that the region responsible for Gfi1-mediated activation lies downstream of TSS. Interestingly, the deletion of a 16-bp nucleotide sequence from the 3′ ends of fragments I and II abolished their activation by Gfi1. We then deleted the various regions downstream of the TSS in the *Hemgn* promoter fragment VI (−70/+63 bp) to more precisely map the region important for Gfi1-mediated activation. Like fragments I and II, deletion of the 16-bp sequence from the 3′ end abrogated its activation by Gfi1 although the 3′ region of 30 bp was required for optimal activation (fragment VIf). Together, these data demonstrated that the +47/+63-bp region of the *Hemgn* promoter is critical for its activation by Gfi1.

Analysis of the 30-bp sequence in the 3′ region utilizing transcription factor binding prediction software (http://tfbind.hgc.jp/) identified potential binding sites for Gfi1, Ikaros, and PU.1. Notably, PU.1 shares its consensus binding motif with Ikaros (38). We introduced mutations in the Gfi1 and Ikaros/PU.1 binding site either alone or in combination in the *Hemgn* promoter fragment II (−567/+63 bp). Interestingly, mutation of either the Gfi1 or Ikaros/PU.1 binding site significantly diminished Gfi1-mediated activation whereas mutation of both binding sites completely abolished its activation by Gfi1, suggesting that Gfi1 may collaborate with Ikaros/PU.1 in the activation of the *Hemgn* promoter.

### Gfi1, Ikaros, and PU.1 bind to the *Hemgn* core promoter

**Figure 4:**
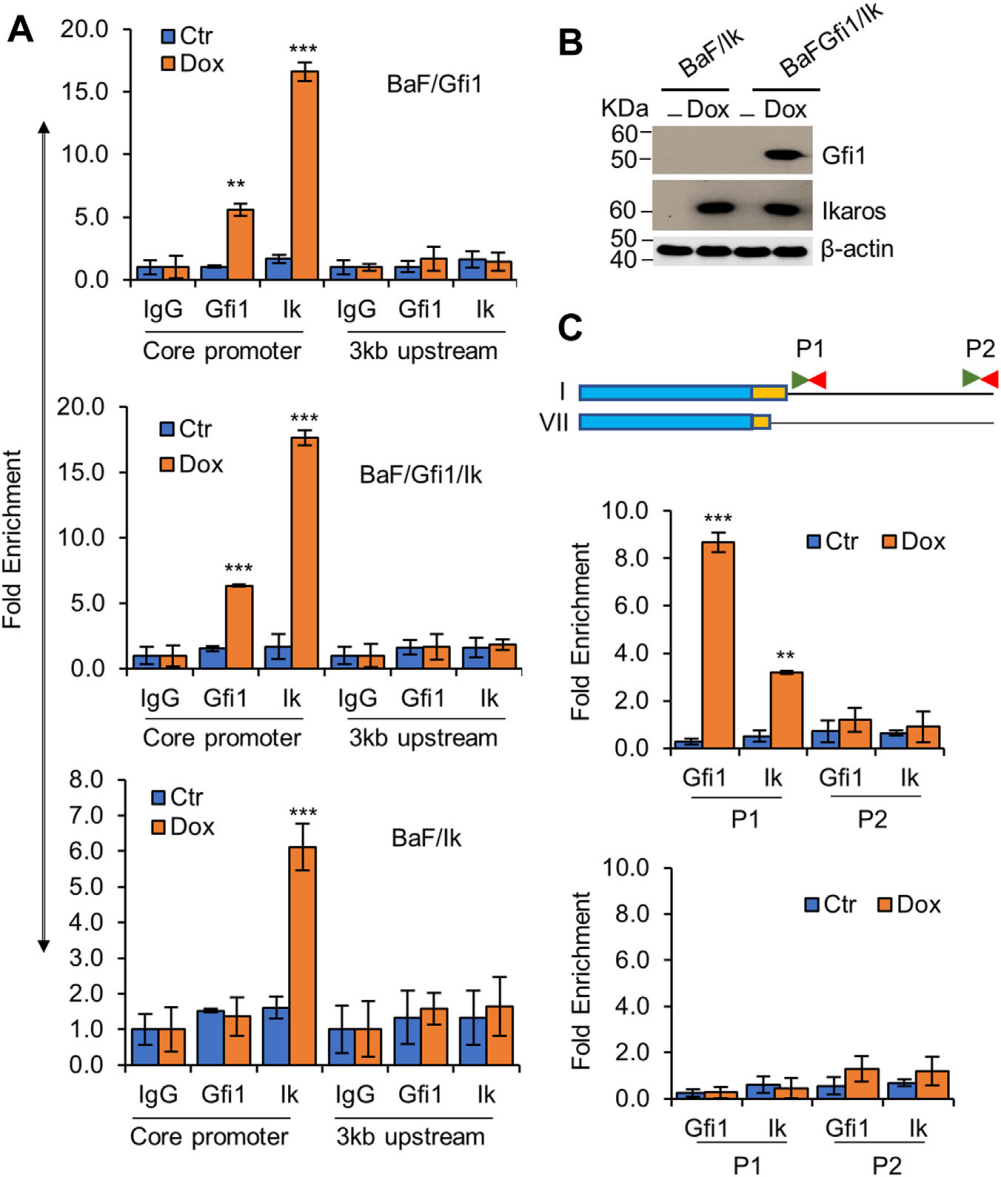
**Gfi1 and Ikaros bind to the *Hemgn* core promoter.***A*, cells as indicated were cultured with and without Dox for 24 h. ChIP assays were performed using antibodies against Gfi1 and Ikaros or IgG as a control, followed by PCR to amplify the *Hemgn* core promoter and the 3-kb upstream region. *B*, expression of Gfi1 and Ikaros in BaF/Ik and BaF/Gfi1/Ik cells. *C*, BaF3/Gfi1/Ik cells were transfected with the reporter plasmid containing fragment I or fragment VII (*upper panel*). The *green* and *red arrows* denote the forward and reverse primers, respectively, used to amplify the proximal (P1) and distal (P2) plasmid sequences. ChIP experiments were conducted as in A using cells transfected with fragment I (*middle panel*) and fragment VII (*bottom panel*). Data are shown as mean ± SD (n = 3). ∗∗*p* < 0.01; ∗∗∗*p* < 0.001.

**Figure 5:**
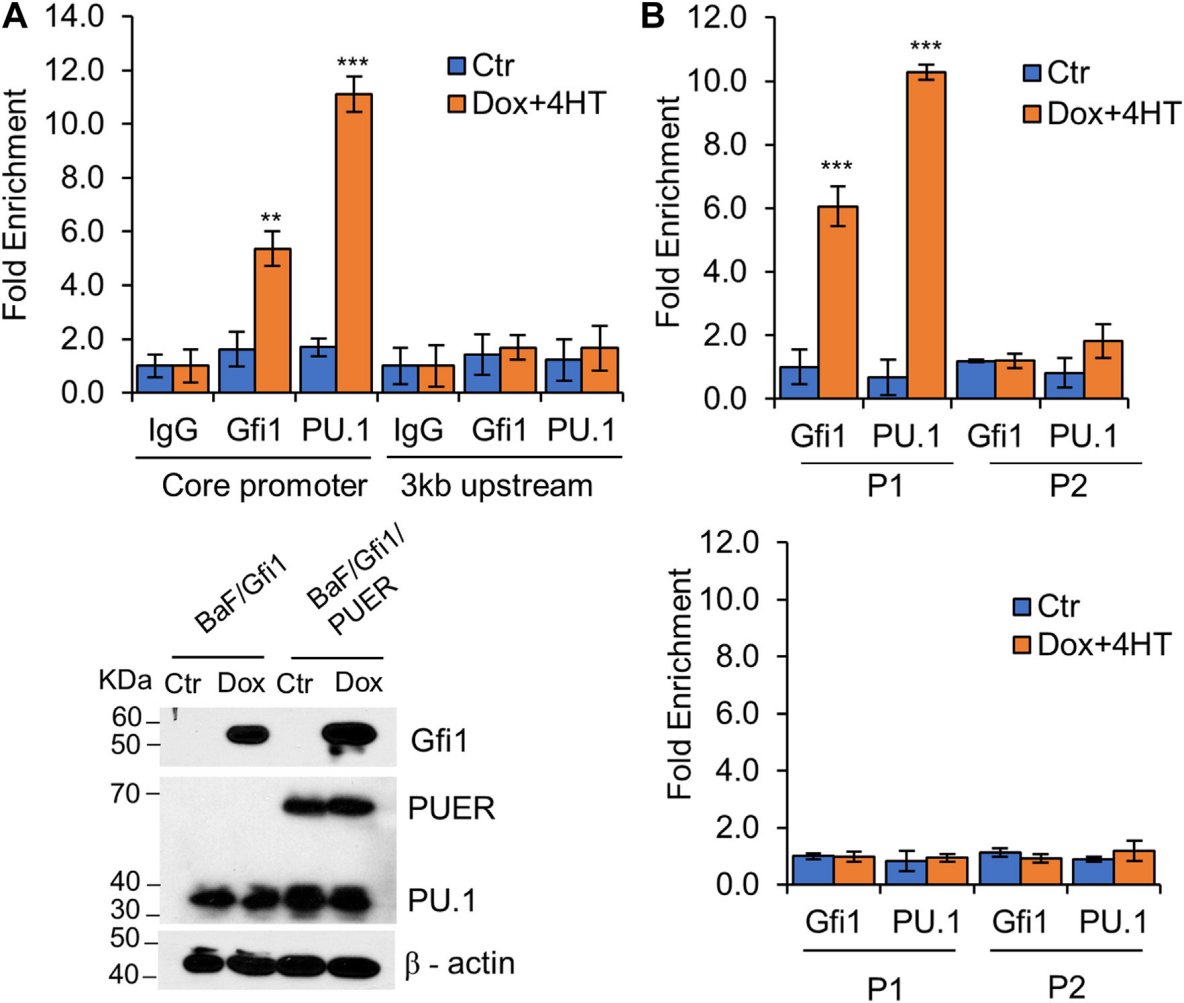
**PU.1 binds to the *Hemgn* core promoter.** BaF/Gfi1/PUER cells were transfected with the reporter plasmid containing fragment I or fragment VII, and cultured without or with Dox (1 μg/ml) and 4-HT (100 nM) for 24 h. ChIP experiments were carried out using antibodies against Gfi1, PU.1 or IgG. *A*, qPCR was conducted to amplify the *Hemgn* core promoter and the 3 kb upstream region in cells transfected with fragment VII (*upper panel*). Expression of PUER along with endogenous PU.1 and Gfi1 was examined (*lower panel*). *B*, the P1 and P2 plasmid sequences were amplified using cells transfected with fragment I (*upper panel*) or fragment VII (*lower panel*). Data are shown as mean ± SD (n = 3). ∗∗*p* < 0.01; ∗∗∗*p* < 0.001.

Chromatin immunoprecipitation (ChIP) assays were then performed to investigate whether Gfi1 and Ikaros bind to the *Hemgn* core promoter in BaF/Gfi1, BaF/Ik, and BaF/Gfi1/Ik cells, which expressed Gfi1, Ikaros, or both in response to Dox (Fig. 4*B*). As shown in Fig. 5*A*, both Gfi1 and Ikaros bound to the *Hemgn* core promoter, but not to the 3-kb upstream region. BaF3 cells expressed a very low level of endogenous Gfi1 that was barely detectable by Western blot analysis (data not shown). It appeared that overexpression of Gfi1 increased Ikaros binding to the *Hemgn* core promoter. On the other hand, Ikaros overexpression had no effect on Gfi1 binding to the *Hemgn* core promoter.

We further conducted plasmid-ChIP experiments to determine whether Gfi1 and Ikaros are specifically bound to the 16-bp (+47/+63 bp) region of the *Hemgn* promoter in BaF/GFI1/Ik cells. The cells were transfected with the reporter plasmid containing either fragment I (−1972/+63 bp) or fragment VII (−1972/+47 bp) lacking the 16 bp region (Fig. 3). After ChIP with antibodies against Gfi1 or Ikaros, the precipitated DNA was analyzed by PCR with primers that amplify the plasmid DNA sequences 215 bp and approximately 2.5 kb (as a negative control) downstream of the *Hemgn* promoter fragments (Fig. 4*C*). Notably, Gfi1 and Ikaros bound to the *Hemgn* promoter fragment I, but not to fragment VII or the plasmid sequence 2.5 kb downstream, demonstrating that they are both specifically bound to the 16-bp region of the *Hemgn* promoter.

To examine whether PU.1 bound to the 16-bp sequence of *Hemgn* promoter, we generated BaF/Gfi1 cells that constitutively expressed PUER in which PU.1 was fused to the estrogen receptor (ER) ligand binding domain, allowing PU.1 to be conditionally activated by 4-hydroxytamaxifen (4-HT). BaF/Gfi1/PUER cells were subsequently transfected with the reporter plasmid containing fragment I or fragment VII and cultured without or with Dox and 4-HT for 24 h, followed by ChIP with the anti-Gfi1 or anti-PU.1 antibody. PCR was then performed to examine the binding of PU.1 and Gfi1 to the endogenous *Hemgn* and to the promoter fragments I/VII with the primers utilized in the above ChIP experiments. As the PCR primers used to amplify the endogenous *Hemgn* core promoter did not amplify fragment VII, Gfi1 and PU.1 binding to endogenous *Hemgn* were examined in cells transfected with fragment VII. As shown in Fig. 5, like Gfi1 and Ikaros, PU.1 bound to the *Hemgn* core promoter and the 16-bp sequence was required for its binding.

Together, these results suggested a potential role of Ikaros and PU.1 in the regulation of Hemgn expression; however, it remained to be determined whether they are involved in Gfi1-mediated Hemgn upregulation.

### Ikaros weakly activates Hemgn but is not essential for Hemgn upregulation by Gfi1

**Figure 6:**
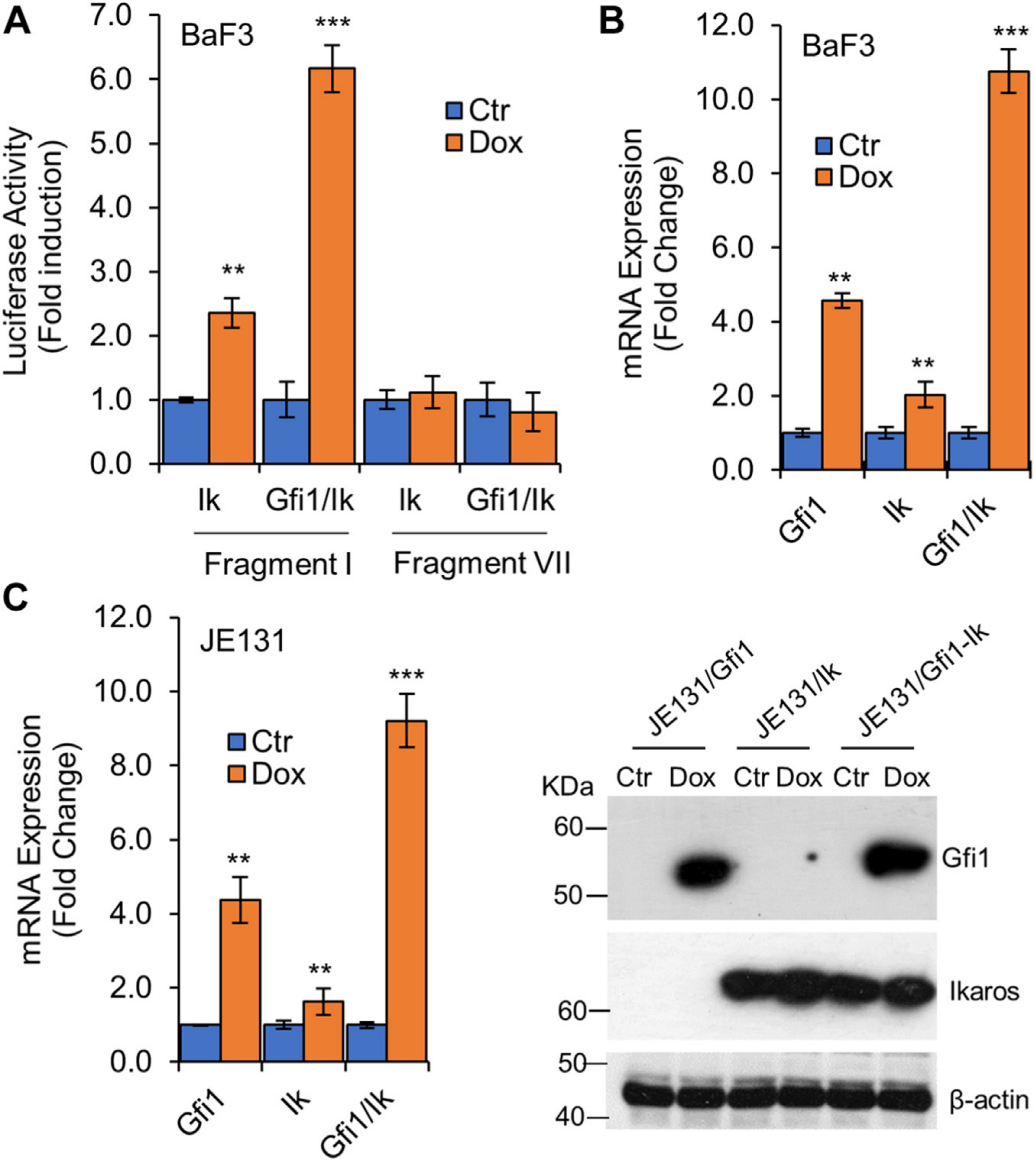
**Ikaros collaborates with Gfi1 to activate *Hemgn* promoter.***A*, BaF/Ik and BaF/Gfi1/Ik cells were transfected with reporter plasmids containing fragment I or VII and then cultured without (Ctr) or with Dox (1 μg/ml) for 24 h prior to examination of luciferase activity. *B*, Hemgn mRNA levels were examined in BaF/Gfi1, BaF/Ik and BaF/Gfi1/Ik cells cultured in the absence (Ctr) or presence of Dox for 24 h. *C*, Hemgn mRNA levels were examined in JE131/Gfi1, JE131/Ik and JE131/Gfi1/Ik cells untreated or treated with Dox for 24 h (*left panel*). Data are shown as mean ± SD (n = 3). ∗∗*p* < 0.01; ∗∗∗*p* < 0.001. Expression of Gfi1 and Ikaros proteins was confirmed by Western blot analysis (*right panel*).

We first investigated the role of Ikaros in the regulation of Hemgn expression. BaF/Ik and BaF/Gfi1/Ik cells were transiently transfected with the reporter plasmid containing fragment I or fragment VII (Fig. 3*A*). Dox treatment resulted in a two-fold increase in the activity of fragment I, but not fragment VII in BaF/Ik cells (Fig. 6*A*). Interestingly, fragment I, but not fragment VII, was strongly activated (6 fold) upon Dox treatment of BaF/Gfi1/Ik cells. Consistent with promoter activation, Hemgn expression was upregulated by approximate two folds in BaF/Ik cells, more than 4.5 folds in BaF/Gfi1 cells, and about 10.8 folds in BaF/Gfi1/Ik cells following Dox treatment (Fig. 6*B*). To address whether Ikaros was required for Hemgn upregulation by Gfi1, we introduced the Dox-inducible Gfi1 into the mouse Ikaros null JE131 T cells (JE131/Gfi1). Notably, Hemgn expression was still upregulated by Gfi1 in the absence of Ikaros (Fig. 6*C*). Constitutive restoration of Ikaros expression in JE131/Gfi1 cells (JE131/Gfi1/Ik) modestly increased Hemgn expression, but significantly augmented Gfi1-mediated Hemgn upregulation. Together, these results indicated that Ikaros is not required for Gfi1-mediated Hemgn upregulation although it had a weak positive effect on, and collaborated with Gfi1 to enhance Hemgn expression.

### PU.1 represses *Hemgn* and is required for Hemgn upregulation by Gfi1

**Figure 7:**
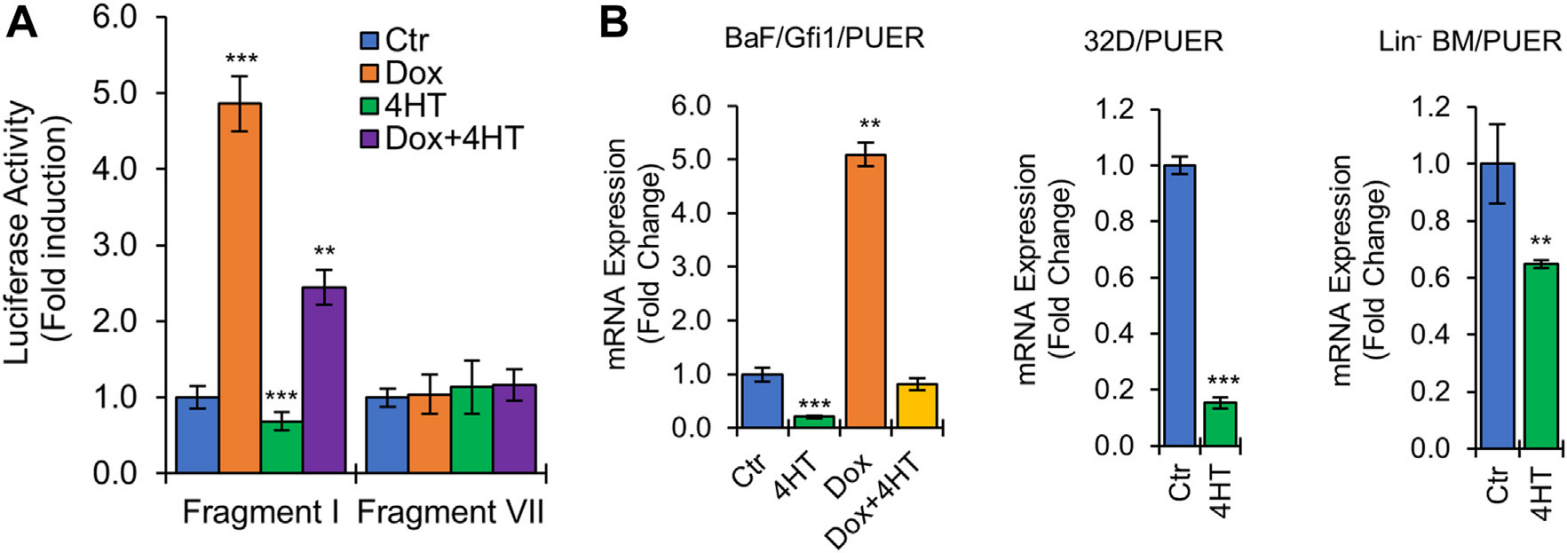
**PU.1 represses *Hemgn* and inhibits Gfi1-mediated activation of *Hemgn*.***A*, BaF/Gfi1/PUER cells were transfected with reporter plasmids containing fragment I or VII and cultured without or with Dox (1 μg/ml), 4HT (100 μM) or both for 24 h prior to evaluation of luciferase activity. *B*, BaF/Gfi1/PUER, 32D and Lin^-^ BM cells transduced with PUER retroviral construct were cultured without or with 4HT, Dox or both as indicated for 24 h prior to examination of Hemgn mRNA levels by qRT-PCR. Data are shown as mean ± SD (n = 3). ∗∗*p* < 0.01; ∗∗∗*p* < 0.001.

We next focused on the role of PU.1 in the regulation of Hemgn expression. In luciferase reporter assay, activation of PU.1 with 4-HT repressed the activity of *Hemgn* promoter fragment I in BaF/Gfi1/PUER cells irrespective of Dox treatment but had no effect on fragment VII (Fig. 7*A*), suggesting that the +47/63 bp region of the *Hemgn* promoter is critical for PU.1-mediated repression. Significantly, 4-HT treatment markedly downregulated Hemgn mRNA expression and completely abolished Gfi1-mediated Hemgn upregulation (Fig. 7*B*). As in BaF3 cells, PU.1 downregulated Hemgn expression in murine myeloid 32D cells and Lin^-^ BM cells.

**Figure 8:**
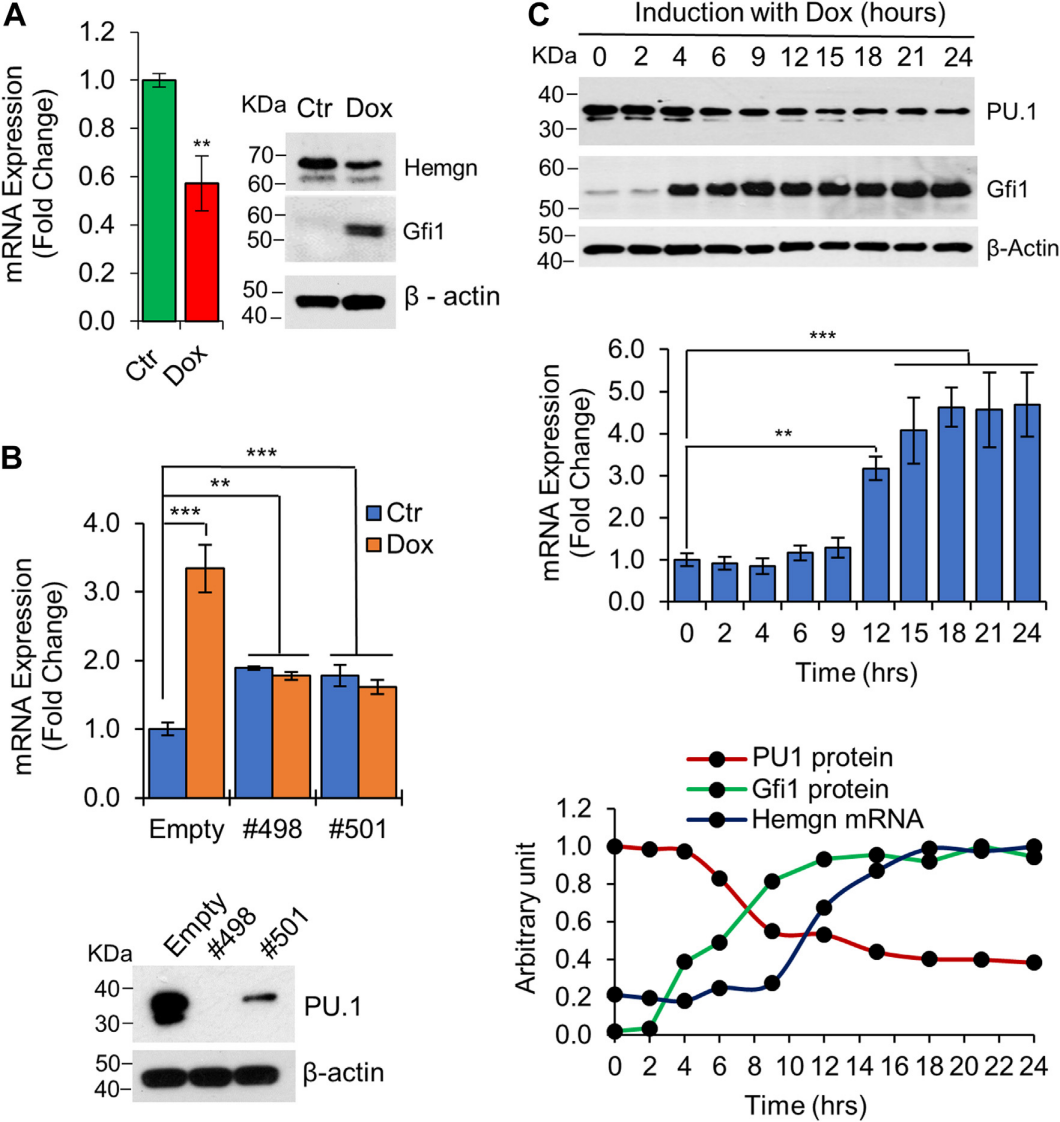
**PU.1 is required for Gfi1-mediated upregulation of Hemgn.***A*, Hemgn mRNA (*left panel*) and protein (*right panel*) levels were examined in PUER/Gfi1 cells untreated or treated with Dox (1 μg/ml) for 24 h. *B*, BaF/Gfi1 cells were transduced with empty or two different PU.1 shRNA-expressing lentiviruses (498 and 501) and examined for Hemgn expression following Dox induction for 24 h (*upper panel*). PU.1 knockdown was confirmed by Western blot analysis (*lower panel*). *C*, BaF/Gfi1 cells were treated with Dox for the indicated times and examined for expression of Gfi1 and PU.1 proteins (*upper panel*) and Hemgn mRNA (*middle panel*). *Lower panel*: Graphical presentation of Gfi1, PU.1, and Hemgn expression. The levels of Gfi1 and PU.1 proteins were based on the densities of bands determined using Image J software. Data are shown as mean ± SD (n = 3). ∗∗*p* < 0.01; ∗∗∗*p* < 0.001.

Gfi1 has been shown to repress *PU.1* and inhibit PU.1 function through direct protein-protein interaction (5, 34). Consistent with these studies, Gfi1 downregulated PU.1 expression in BaF/Gfi1 cells at both mRNA and protein levels (Fig. S1*A*). In contrast, PU.1 mRNA was significantly increased in Lin- BM cells from *Gfi1*^*−/−*^ mice (Fig. S1*B*). To address whether Gfi1 upregulated Hemgn through repressing PU.1, we introduced the Dox-inducible Gfi1 into myeloid PUER cells, which were derived from *PU.1*^*−/−*^ mice and transduced with PUER. In the absence of 4-HT, PUER is inactive. As shown in Fig. 8*A*, treatment of PUER cells with Dox downregulated rather than upregulated Hemgn. We further evaluated the effect of PU.1 knockdown on Hemgn upregulation by Gfi1. The expression of PU.1 was knocked down in BaF/Gfi1 cells using two different PU.1 shRNAs (Fig. 8*B*). PU.1 knockdown resulted in augmented expression of Hemgn mRNA but abolished Gfi1-mediated Hemgn upregulation.

If Gfi1 upregulated Hemgn through repressing PU.1, downregulation of PU1 should occur before Hemgn upregulation. We therefore examined the dynamics of Gfi1, PU.1 and Hemgn expression in BaF/Gfi1 cells following Dox treatment. Gfi1 protein expression was induced as early as 4 h following Dox induction whereas PU.1 downregulation occurred at approximately 6 to 9 h (Fig. 8*C*). Notably, upregulation of Hemgn mRNA was observed at 12 h post Dox treatment. Thus, PU.1 downregulation by Gfi1 correlated well with Hemgn upregulation in BaF/Gfi1 cells. Taken together, these results revealed an indirect regulatory mechanism of Hemgn upregulation by Gfi1, *i.e.*, through repression of *PU.1*.

### Hemgn has a role in Gfi1-mediated protection against DNA damage-induced apoptosis

**Figure 9:**
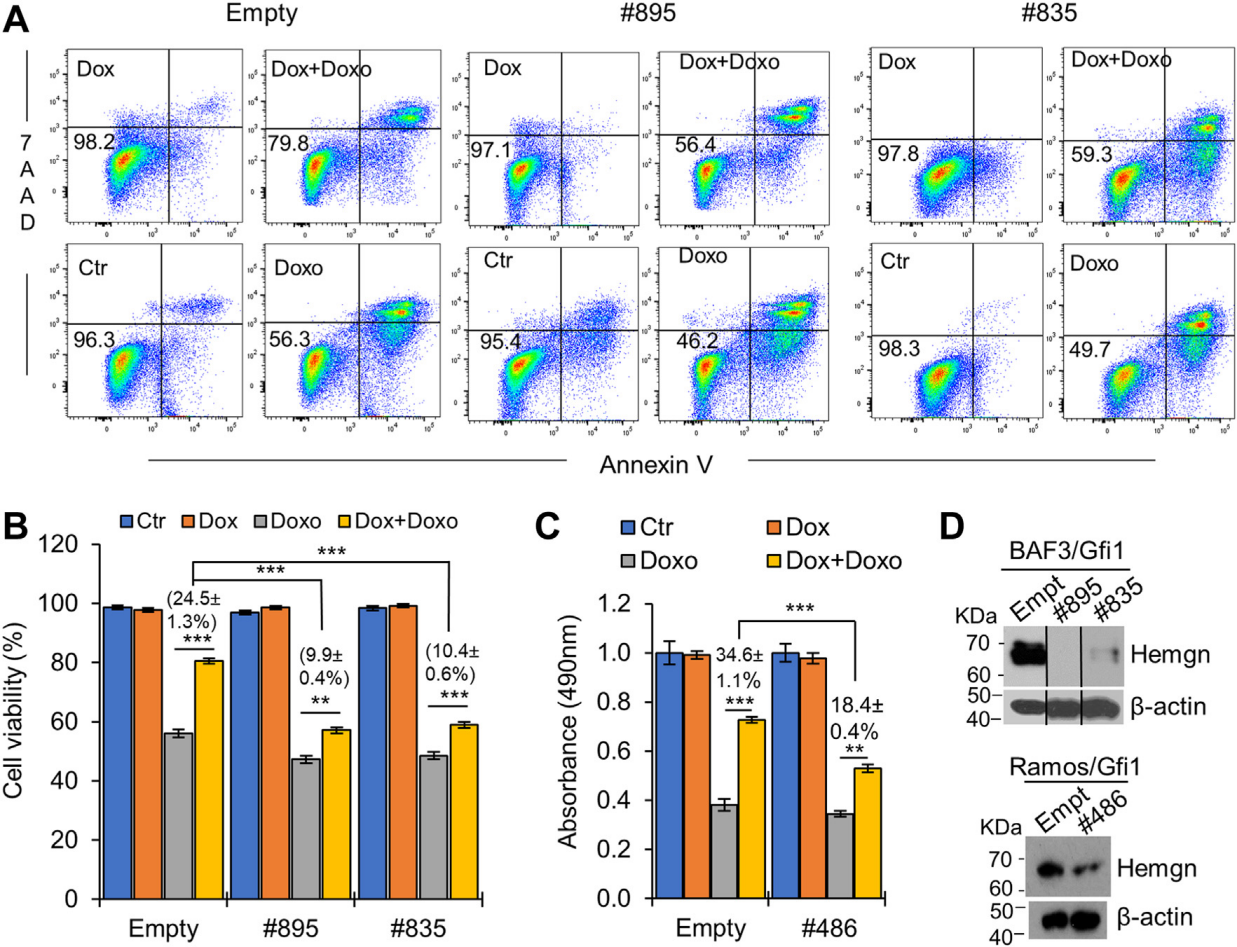
**Knockdown of Hemgn diminishes the protective effect of Gfi1 on DNA damage-induced apoptosis.** BaF/Gfi1 and Ramos/Gfi1 cells were transduced with empty or Hemgn shRNA-expressing lentiviral constructs, followed by treatment with Dox (1 μg/ml) for 6 h prior to treatment with Doxo (200 ng/ml for BaF/Gfi1 cells and 2 mg/ml for Ramos/Gfi1 cells) for 24 h. *A*, the apoptosis of BaF/Gfi1 cells was examined by Annexin V assay. Shown is a representative flow cytometry experiment. The numbers denote percentages of live cells. *B*, Data from 3 independent flow cytometry experiments are presented. *C*, live Ramos/Gfi1 cells were quantitated using MTS assay. The numbers in (*B*) and (*C*) indicate increases in the viability of Doxo-treated cells cultured in the presence *versus* absence of Dox, and are shown as mean ± SD (n = 3). ∗∗*p* < 0.01; ∗∗∗*p* < 0.001. *D*, Hemgn expression was examined by Western blot analysis.

Gfi1 has been shown to inhibit apoptosis induced by DNA damage in hematopoietic cells in part through post-translational modifications at the C-terminal domain of the p53 protein, leading to suppression of p53 activity (39). However, our previous study showed that Gfi1 also inhibited DNA damage-induced apoptosis in p53-deficient hematopoietic cells (18). Notably, it has been shown that Hemgn was induced in response to DNA damage and inhibited DNA damage-induced apoptosis (24, 36). We examined whether the protective activity of Gfi1 is mediated in part through Hemgn. The expression of Hemgn in BaF/Gfi1 cells was knocked down using two different shRNAs (Fig. 9*D*). A shRNA against human HEMGN was utilized to knock down its expression in the p53-deficient Ramos/Gfi1 cells. The cells were then cultured without or with Dox for 6 h followed by treatment with Doxo for 24 h. Apoptosis was evaluated by Annexin V staining for BaF/Gfi1 cells and by MTS assay for Ramos/Gfi1 cells. MTS assay instead of annexin V staining was used because Ramos/Gfi1 cells were treated with Doxo at 2 mg/ml, which interfered with flow cytometric analysis of annexin V-stained cells (18). Doxo treatment decreased the viabilities of BaF/Gfi1 and Ramos/Gfi1 cells (Fig. 9). Induction of Gfi1 expression with Dox led to an increase in cell viability by 24.5% in Doxo-treated BaF/Gfi1 cells and by 34.6% in Ramos/Gfi1 cells (Fig. 9, *B* and *C*). However, the increases in cell viability following Dox-induction of Gfi1 expression dropped to approximately 10% and 18% in BaF/Gfi1 and Ramos/Gfi1 cells, respectively, upon knockdown of Hemgn expression. To further demonstrate that suppression of DNA damage-induced apoptosis by Hemgn was independent of p53, we transduced Ramos cells with the Hemgn-expressing retroviral construct. As shown in Fig. S2, Hemgn overexpression inhibited apoptosis of Ramos cells treated with Doxo.

**Figure 10:**
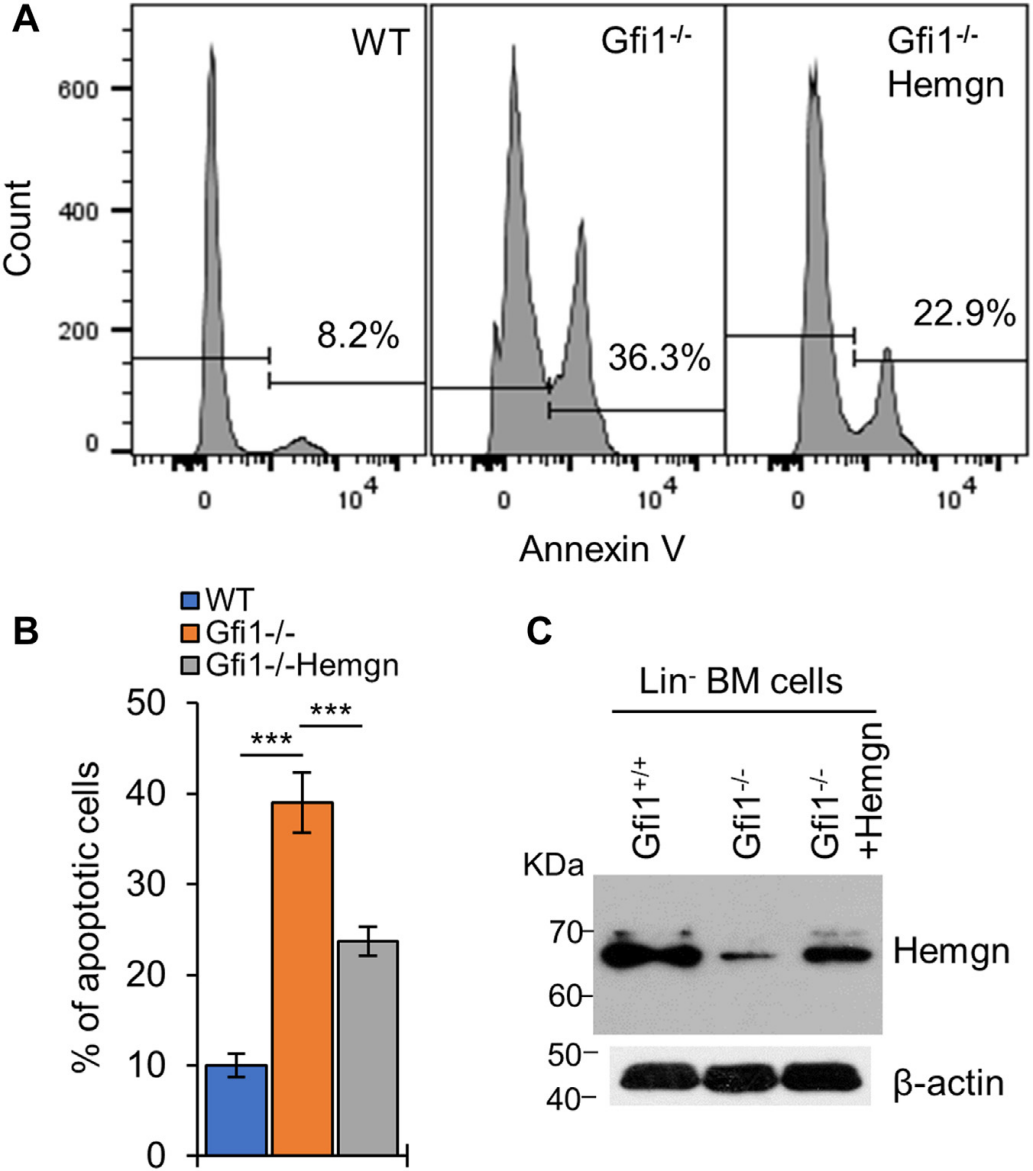
**Restoration of Hemgn expression in *Gfi1***^***−/−***^**BM cells partially rescues the hypersensitivity to DNA damage.** Lin^−^ cells were isolated from WT and *Gfi1*^*−/−*^ mice. *Gfi1*^*−/−*^ cells were transduced with the empty or Hemgn-expressing retrovirus and sorted based on GFP. Cells were then treated with Doxo (50 ng/ml) for 24 h and examined for apoptosis by flow cytometry based on annexin V staining. *A*, shown is a representative experiment of flow cytometry. *B*, data from 3 independent flow cytometry experiments are presented. Data are shown as mean ± SD (n = 3). ∗∗*p* < 0.01; ∗∗∗*p* < 0.001. *C*, expression of Hemgn protein in the different cell populations was examined by Western blot analysis.

BM cells from *Gfi1*^*−/−*^ mice showed increased sensitivity to DNA damage-induced apoptosis (4). As Hemgn expression in Gfi1^−/−^ BM cells was decreased (Fig. 1*C*), we assessed whether restoration of Hemgn expression rescued the hypersensitivity of Gfi1^−/−^ BM cells to DNA damage. Lin^-^ BM cells from *Gfi1*^*−/−*^ mice were transduced with an empty retroviral construct or Hemgn-expressing construct and sorted for GFP expression 48 h later. Hemgn expression was partially restored in the sorted BM cells (Fig. 10*C*). After treatment with Doxo, apoptosis was evaluated by Annexin V staining. The percentages of Annexin V-positive (apoptotic) cells were approximately 10% and 39% for WT and *Gfi1*^*−/−*^ BM cells, respectively (Fig. 10*B*). Partial restoration of Hemgn expression in *Gfi1*^*−/−*^ BM cells reduced the percentage of apoptotic cells to about 24%. These results reveal the role of Hemgn in Gfi1-mediated protection against DNA damage-induced apoptosis.

## Discussion

Gfi1 is a transcriptional repressor critically involved in hematopoiesis. Transcriptional repression by Gfi1 is mediated mainly through interaction with LSD1. In this study, we examined whether Gfi1 may function to augment gene expression. We show here that Gfi1 upregulates Hemgn in both mouse and human hematopoietic cells. The +47/63 bp region of *Hemgn* promoter is required for Gfi1-mediated upregulation, which is dependent on its interaction with LSD1. This 16-bp region contains conserved binding sites for Gfi1 and Ikaros/PU.1. Using ChIP assay, we show that all three transcription factors bind to the 16-bp region. However, while Ikaros activates the Hemgn promoter and collaborates with Gfi1 to augment Hemgn expression, it is not required for Gfi1-mediated upregulation of Hemgn. In contrast, PU.1 represses Hemgn and blocks Gfi1-mediated Hemgn upregulation. Significantly, Gfi1 is unable to upregulate Hemgn in PU.1-knocked down and PU.1-deficient cells. In line with previous studies (5, 34), we show that Gfi1 represses PU.1 and that Gfi1-mediated PU.1 downregulation precedes Hemgn upregulation. These results are consistent with a mode of action in which Gfi1 represses *PU.1*, leading to *Hemgn* derepression.

Hemgn is a nuclear protein that is primarily expressed in HSCs and early progenitor cells (22, 23). Hemgn overexpression in human and mouse hematopoietic stem and progenitor cells (HSPCs) has been shown to enhance their proliferation, survival, and self-renewal (25, 27, 40, 41). Interestingly, like Gfi1^−/−^ HSCs, Hemgn^−/−^ HSCs are also impaired in their ability to reconstitute hematopoiesis in lethally irradiated recipient mice (24). An important function of Gfi1 is to inhibit stress-induced apoptosis, which is important for its role in hematopoiesis, in particular for maintaining the functional integrity of HSCs and the development of T lymphocytes (3, 4, 42). The anti-apoptotic activity of Gfi1 is also critical for its oncogenic potential in lymphoid leukemia and lymphoma (43). It has been shown that Gfi1 protects cells from DNA damage-induced apoptosis in part through inhibiting p53 function due to its involvement in post-translational modifications of p53 protein (39). However, our previous study indicated that Gfi1 may inhibit DNA damage-induced apoptosis *via* p53-independent mechanisms (18). In this study, we have shown that Hemgn knockdown partially abolished the protective effect of Gfi1 on DNA damage-induced apoptosis in both p53-expressing and p53-deficient cells. In contrast, partial restoration of Hemgn expression in *Gfi1*^*−/−*^ BM cells diminished the hypersensitivity to DNA damage. It is of note that Hemgn has been shown to be upregulated and phosphorylated in response to DNA damage and inhibit stress-induced apoptosis (24, 33, 36). We have further shown here that Gfi1 and DNA damage act in collaboration to augment Hemgn expression in a p53-independent manner. Our data indicate that Hemgn upregulation contributes to the anti-apoptotic function of Gfi1 and suggests that Hemgn may have an important role in DNA damage response.

Our data also reveal *Hemgn* as a novel PU.1-repressed target gene. Interestingly, Hemgn has been shown to upregulate the expression of PU.1 in HSCs (34). PU.1 is a master regulator of hematopoiesis that is important for self-renewal of HSCs and critically implicated in regulating myeloid and lymphoid development in a graded manner (44, 45, 46, 47, 48). Notably, PU.1 has been shown to function as a potent tumor suppressor in myeloid and B cell lineages (49, 50, 51, 52). A reduction in PU.1 expression to 20% of normal levels induced the development of AML in mice (49). Conversely, increased expression of PU.1 led to growth arrest and apoptosis in leukemic, multiple myeloma, and lymphoma cells (51, 52, 53). The mechanisms by which PU.1 induces growth arrest and apoptosis are incompletely understood but may involve PU.1-mediated downregulation of c-Myc, Bcl-2, and IRF4 (52, 53). We show here that PU.1 represses *Hemgn* by binding to its +47/+63 region. As Hemgn enhances cell proliferation and survival, and its expression is increased in AML and ALL cells, it would be interesting to examine whether repression of *Hemgn* contributes to the role of PU.1 as a tumor suppressor.

We further show here that contrary to PU.1, Ikaros transcriptionally activates *Hemgn* and collaborates with Gfi1 to upregulate Hemgn expression. Interestingly, Ikaros has been shown to collaborate with Gfi1 to activate gene expression by co-occupying the regulatory regions of target genes, including *NOTCH3* (54). However, Ikaros deficiency did not affect Gfi1-mediated Hemgn upregulation, indicating that Ikaros is not required for Hemgn upregulation by Gfi1. Like PU.1, Ikaros is a master regulator of hematopoiesis (55, 56, 57) and functions as an important tumor suppressor in the hematopoietic system, particularly in B cell lineage (57, 58, 59). Interestingly, in contradiction to its role as a tumor suppressor, immunomodulatory imide drugs (IMiDs), which induce Ikaros degradation resulting in reduced proliferation and survival of cancer cells, are frequently used in combination therapies for patients with multiple myeloma, lymphoma, and certain forms of myelodysplastic syndrome (57, 59). It appears that the activity of Ikaros may depend on cell types and developmental stages. For instance, Ikaros is involved in transcriptional repression of the CDK inhibitor Cdkn1a encoding p21WAF1/CIP1 in certain hematopoietic cells (57). It is tempting to evaluate whether Ikaros may positively regulate cell proliferation and survival by transcriptionally activating *Hemgn* in certain cells.

## Experimental procedures

### Cell lines and cell culture

The maintenance of murine pro-B Ba/F3, myeloid 32D, and human Burkitt lymphoma cell lines has been described before (18, 35, 60). Murine Ikaros-null T-cell leukemia cell line JE131 (61) was cultured in RPMI-1640 medium supplemented with 10% fetal bovine serum (FBS) and 1% penicillin/streptomycin (P/S). PUER cells (62) were kindly provided by Dr Richard Dahl (Indiana University School of Medicine) and cultured in RPMI-1640 medium with 10% charcoal-stripped FBS and 1% P/S. Ba/F3, 32D and Ramos cells transduced with the Dox-inducible lentiviral expression construct pTMPrtTA-Gfi1-GFP encoding Gfi1 and GFP have been described before (18, 35, 63).

### Expression constructs and stable gene delivery

The Dox-inducible Ikaros-expressing vector pTMPrtTA-Ik-GFP was generated by replacing the Gfi1 cDNA in pTMPrtTA-Gfi1-GFP with the Ikaros cDNA (61), kindly provided by Dr Andrew Wells (The Children’s Hospital of Philadelphia), and then introduced into Ba/F3 cells through lentiviral transduction as described (18, 63) to generate BaF/Ik cells. The GFP cDNA in pTMPrtTA-Ik-GFP was replaced with RFP cDNA to generate pTMPrtTA-Ik-RFP, which was then transduced into BaF/Gfi1 cells expressing the Dox-inducible Gfi1 (BaF/Gfi1) to generate BaF/Gfi1/Ik cells. The Dox-inducible pLentiGS-minCMV-TET-puromycin constructs containing Gfi1-LSD1 or Znf-LSD1 cDNA (64) were kindly provided by Dr Tim Somervaille (Cancer Research UK Manchester Institute, The University of Manchester) and transduced into Ba/F3 cells, followed by puromycin selection (2 μg/ml). For generation of JE131 cells reconstituted with Ikaros and expressing Dox-inducible Gfi1, cells were first transduced with pTMPrtTA-Gfi1-GFP and then with MIGR1-Ikaros (65). PUER cells expressing the Dox-inducible Gfi1 (PUER/Gfi1) were generated by lentiviral transduction with pTMPrtTA-Gfi1-GFP.

### Construction of luciferase promoter constructs and luciferase reporter assay

A murine *Hemgn* promoter fragment spanning from −1972 bp to +63 bp (fragment I; see Fig. 3*A*) was amplified from mouse genomic DNA and cloned into the MluI and XhoI sites of the pGL3 basic vector (Promega, Madison, WI). The other *Hemgn* promoter fragments were derived from fragment I using the PCR-base technique. Mutations in *Hemgn* promoter fragment II were generated by site-directed mutagenesis using the Quick-change site-directed mutagenesis kit (Stratagene). The nucleotide sequences of all promoter fragments and mutants were confirmed by DNA sequencing. Ba/F3 cells were transfected with the reporter constructs containing the different *Hemgn* promoter fragments by electroporation. Approximately 2 hours post-transfection, cells were either left untreated or treated with Dox (1 μg/ml) and/or 4-HT (100 nM). Luciferase activities were measured 24 h post-transfection using a Molecular Devices Lmax luminometer. All luciferase values are shown as mean ± SD of three independent experiments.

### Real-time reverse transcription polymerase chain reaction (qRT-PCR)

Total RNA was extracted with TRIzol reagent (ABP Biosciences) and reverse transcribed into cDNAs using the LunaScript RT SuperMix Kit (NEB) according to the manufacturer's instructions. The relative mRNA levels of various genes were quantified by qRT-PCR using iTaq Universal SYBR Green Supermix (Bio-Rad) and normalized to GAPDH mRNA expression as described previously (60).

### Chromatin immunoprecipitation (ChIP assay) and plasmid ChIP assay

Cells were fixed with 1% formaldehyde for 10 min to stabilize protein-DNA complexes, followed by the addition of 0.125 M glycine to terminate the cross-linking process. Subsequent steps were performed using the Simple ChIP Enzymatic Chromatin IP Kit according to the manufacturer's protocol (Cell Signaling Technology). Immunoprecipitation (IP) was carried out using antibodies against Gfi1, Ikaros, PU.1, or normal rabbit IgG. The immunoprecipitated DNA was purified and quantified by qPCR as described above using primers listed in Table S1. For plasmid ChIP assay, cells were transfected with the reporter plasmids containing *Hemgn* fragment I (1972/+63 bp) or fragment VI (−1972/+47 bp) and treated with Dox and/or 4HT as described above. ChIP experiments were carried out as described above with qPCR performed using the primers shown in Table S1 to amplify the plasmid P1 and P2 regions (see Fig. 4*C*).

### Mice and bone marrow cell isolation

Mice used in the experiments were bred and housed at the animal facility of The University of Toledo. All experiments involving mouse bone marrow (BM) cells were conducted in compliance with the guidelines approved by the Institutional Animal Care and Use Committee (IACUC) of The University of Toledo and were carried out according to the approved protocol. BM cells were isolated from six- to 8-week-old C57BL/6 *Gfi1*^*+/+*^ and *Gfi1*^*−/−*^ mice (7) as previously described (66). Lin^−^ cells were purified using the mouse Lineage Cell Depletion kit (Miltenyi Biotec) according to the manufacturer’s protocol and were cultured in IMDM media supplemented with 20% BIT9500 (Stemcell Technologies), 10 ng/ml IL-3, 20 ng/ml IL-6, and 25 ng/ml stem cell factor (SCF; Peprotech).

### RNA interference

Ba/F3 or Ramos cells were infected with the pLKO.1 lentiviral construct containing short hairpin RNAs (shRNAs) against mouse and human Hemgn and selected in 2 μg/ml puromycin 48 h later as previously described (18, 63). The lentiviral constructs containing shRNAs targeting human Hemgn (67) (clone ID: TRCN0000430486) and mouse Hemgn (clone IDs: TRCN0000174895 and TRCN0000193835) were purchased from Sigma Aldrich. The lentiviral constructs containing shRNAs targeting mouse PU.1 (Clone IDs: TRCN0000009498 and TRCN0000009501) were obtained from Horizon Discovery.

### Western blot analysis

Cells were lysed in SDS lysis buffer containing 1% SDS, 50 mM Tris–HCl (pH 8.0), and 10 mM EDTA (pH 8.0) as previously described (68). Proteins were separated by SDS-PAGE before being transferred onto polyvinylidene difluoride (PVDF) membranes. The membranes were then incubated with the appropriate antibodies, and signals were detected using enhanced chemiluminescence. The antibodies against Gfi1 (N-20), PU.1 (C-3), Hemogen (G-2), and Rabbit IgG were purchased from Santa Cruz Biotechnology. Anti-Ikaros antibody (D10E5) and monoclonal ANTI-FLAG M2 antibody were purchased from Cell Signaling Technology and Sigma-Aldrich, respectively. Anti-β-actin antibody (Cat#: 66,009–1) was obtained from Proteintech.

### Apoptosis assay

Apoptosis was assessed using the Annexin V-PE apoptosis detection kit (BD Biosciences) according to previously established methods (69). In brief, 0.3 × 10^6^ cells were collected and stained with Annexin V-PE and 7-amino-actinomycin (7-AAD). The cells were then analyzed by two-color flow cytometry using LSRFortessa (BD Biosciences) as previously described (18).

### MTS assay

The experiments were conducted using the CellTiter 96 AQueous One Solution Cell Proliferation Assay kit following the manufacturer's instructions (Promega, Madison, WI), as described previously (18). Ramos cells (2 × 10^4^) were plated in triplicate in 100 μl of RPMI 1640 medium in 96-well plates, with or without Doxorubicin (2 mg/ml) for 24 h. CellTiter 96 AQueous One Solution Reagent was added to each well, and incubated for 4 h. The absorbance was measured at a wavelength of 490 nm using a Lmax luminometer (Molecular Devices, Sunnyvale, CA).

### Statistics

Statistical analysis was performed using a two-tailed student’s *t* test to compare the differences between any pair of data and to calculate the *p*-values. The data are depicted as mean ± SD in the figures. A *p*-value < 0.05 was considered significant and denoted by ∗, while *p* < 0.01 was represented by ∗∗, and *p* < 0.001 by ∗∗∗.

## Data availability

The data generated in this study are available upon reasonable request from the corresponding authors.

## Supporting information

This article contains supporting information.

## Conflict of interest

The authors declare that they have no conflicts of interest with the contents of this article.
