# Supplementary material for: Upregulation of nuclear protein Hemgn by transcriptional repressor Gfi1 through repressing PU.1 contributes to the anti-apoptotic activity of Gfi1
Source: J Biol Chem. 2024 Oct 5;300(11):107860. doi: 10.1016/j.jbc.2024.107860 (PMC11550643; doi:10.1016/j.jbc.2024.107860)
Supplement: Supporting information [file mmc1.pdf]

Supporting information

Upregulation of nuclear protein Hemgn by transcriptional repressor Gfi1 through repressing PU.1 contributes to the anti-apoptotic activity of Gfi1

Binod G C[1], Laney Jia Hoyt[1], Sinisa Dovat[2] and Fan Dong[1]

List of material included: figures S1 and S2, and table S1

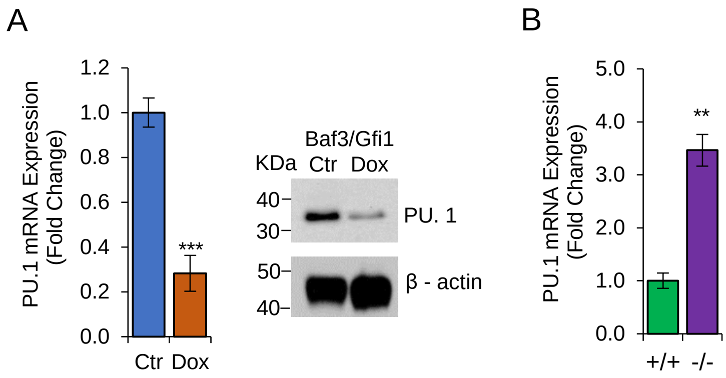

Figure S1. Gfi1 represses *PU.1*. (A) BaF/Gfi1 cells were untreated (Ctr) or treated with Dox for 24 hours, followed by evaluating the expression of PU.1 mRNA using qRT-PCR (left) and PU.1 protein by Western blot analysis (right). (B) PU.1 mRNA expression in Lin^- BM cells from $Gfi1^{+/+}$ and $Gfi1^{-/-}$ mice was examined by qRT-PCR. Data are shown as mean ± SD (n = 3). $**p < 0.01$; $***p < 0.001$.

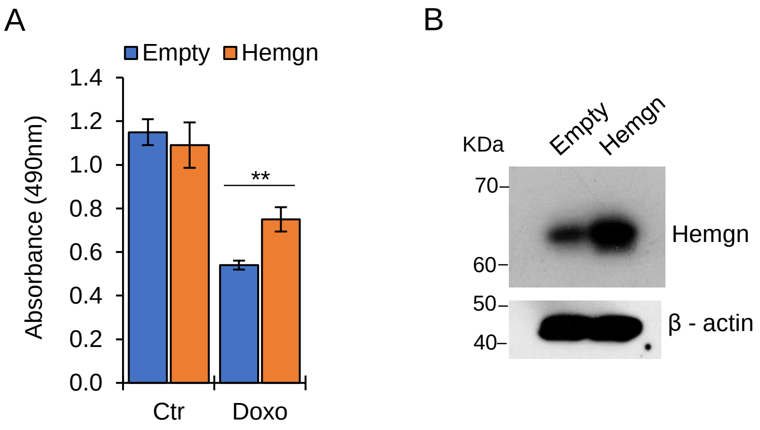

Figure S2. Overexpression of Hemgn protects Ramos cells against DNA damage-induced apoptosis. Cells were transduced with empty or a Hemgn-expressing retroviral construct, Live Ramos cells were quantitated using MTS assay. Data are shown as mean ± SD (n = 3). $***p < 0.001$. (B) Hemgn expression was examined by Western blot analysis.

Table S1. The nucleotide sequences of primers used in this study

Primer Name	Sequence (5’-3’)
m-Hemgn-RT-FW	ACAACTGAGGAAGAGAAAAGCTG
m-Hemgn-RT-RV	CCTGTTCTCTGATACTTGCGTTT
hu-Hemgn-RT-FW	AGGATGTGCCTAAAGAATGCT
hu-Hemgn-RT-RV	ATTCCTGGGTCTTCTTTGGG
m-PU.1-RT-FW	ACCAACGTCCAATGCATGAC
m-PU.1-RT-RV	GCATCTGTTCCAGCTCCATG
m-GAPDH FW	TTCACCATGGAGAAGGC
m-GAPDH RV	GGCATGGACTGTGGTCATGA
hu-GAPDH FW	CGACCACTTTGTCAAGCTCA
hu-GAPDH RV	AGGGGAGATTCAGTGTGGTG
pGL3-b-ChIP-FW (P1)	CTTGGCATTCCGGTACTGTT
pGL3-b-ChIP-RV (P1)	AGGAACCAGGGCGTATCTCT
pGL3-b-ChIP-FW (P2)	AGTTCGGTGTAGGTCGTTCG
pGL3-b-ChIP-RV(P2)	GTCTTACCGGGTTGGACTCA
HemgnChIP3kb up FW	GTACGCTAGGGCTTGCAGAA
HemgnChIP3kb up RV	GGAAGTCAGGCAAGGTGTCT
Hemgn ChIP core-p FW	CCATTTCCCCCTCACACCTG
Hemgn ChIP core-p RV	GTTTGCTTGTCACTTCCCGC
